# New treatments for rare bone diseases: hypophosphatemic rickets/osteomalacia

**DOI:** 10.20945/2359-3997000000555

**Published:** 2022-11-10

**Authors:** Julia Vieira Oberger Marques, Carolina Aguiar Moreira, Victoria Zeghbi Cochenski Borba

**Affiliations:** 1 Certificação Board em Endocrinologia e Metabolismo pela Sociedade Brasileira de Endocrinologia e Metabologia Residência em Endocrinologia no Serviço de Endocrinologia do Hospital de Clínicas da Universidade Federal do Paraná Curitiba PR Brasil Residência em Endocrinologia no Serviço de Endocrinologia do Hospital de Clínicas da Universidade Federal do Paraná (SEMPR/UFPR); Certificação Board em Endocrinologia e Metabolismo pela Sociedade Brasileira de Endocrinologia e Metabologia (SBEM); Medicina Interna pela UFPR, Curitiba, PR, Brasil; 2 Universidade Federal do Paraná Centro Acadêmico de Pesquisa do Instituto Pró-Renal Departamento de Clínica Médica Curitiba PR Brasil Departamento de Clínica Médica da Universidade Federal do Paraná (UFPR); Centro Acadêmico de Pesquisa do Instituto Pró-Renal (PRO), Curitiba, PR, Brasil; 3 Universidade Federal do Paraná Unidade de Ossos do Serviço de Endocrinologia Departamento de Clínica Médica Arkansas PR Brasil Departamento de Clínica Médica da Universidade Federal do Paraná (UFPR); Unidade de Ossos do Serviço de Endocrinologia do Hospital de Clínicas da UFPR (SEMPR/UFPR), Curitiba, PR, Brasil; Unidade de Ossos da University of Arkansas for Medical Sciences (UAMS), Arkansas, EUA

**Keywords:** Hypophosphatemia, osteomalacia, rickets, FGF-23, burosumab

## Abstract

Phosphorus is one of the most abundant minerals in the human body; it is required to maintain bone integrity and mineralization, in addition to other biological processes. Phosphorus is regulated by parathyroid hormone, 1,25-dihydroxyvitamin D_3_ [1,25(OH)_2_D_3_], and fibroblast growth factor 23 (FGF-23) in a complex set of processes that occur in the gut, skeleton, and kidneys. Different molecular mechanisms – overproduction of FGF-23 by tumors responsible for oncogenic osteomalacia, generation of an FGF-23 mutant that is resistant to cleavage by enzymes, and impaired FGF-23 degradation due to a reduction in or loss of the PHEX gene – can lead to FGF-23-stimulating activity and the consequent waste of urinary phosphate and low levels of 1,25(OH)_2_D_3_. Conventional treatment consists of multiple daily doses of oral phosphate salts and vitamin D analogs, which may improve radiographic rickets but do not normalize growth. Complications of the conventional long-term treatment consist of hypercalcemia, hypercalciuria, nephrolithiasis, nephrocalcinosis, impaired renal function, and potentially chronic kidney disease. Recently, burosumab, an antibody against FGF-23, was approved as a novel therapy for children and adults with X-linked hypophosphatemia and patients with tumor-induced osteomalacia. Burosumab showed good performance in different trials in children and adults. It increased and sustained the serum phosphorus levels, decreased the rickets severity and pain scores, and improved mineralization. It offers a new perspective on the treatment of chronic and disabling diseases. Arch Endocrinol Metab. 2022;66(5):658-65

## INTRODUCTION

Phosphorus is one of the most abundant minerals in the human body and is required, in association with calcium, to maintain bone integrity and mineralization. The maintenance of intracellular and extracellular phosphate levels is important for many biological processes, including energy metabolism, cell signaling, regulation of protein synthesis, skeletal development, and bone integrity. Phosphorus is regulated by a complex set of processes that occur in the gut, skeleton, and kidneys (1).

In the skeleton, phosphate is primarily complexed with calcium in the form of hydroxyapatite crystals; the remaining phosphate appears as amorphous calcium phosphate. Maintaining phosphorus levels is also important to allow apoptosis of the chondrocytes in the growth plate with subsequent mineralization. If there is not enough phosphorus, the chondrocytes do not go into apoptosis, and osteoid accumulates with If there is not enough phosphorus, the chondrocytes do not go into apoptosis, and osteoid accumulates without mineralization, resulting in growth retardation, rickets, and osteomalacia (2).

Hypophosphatemia will occur when there is inadequate phosphorus intake, an absorption deficit in the intestine, or excessive renal wasting as a result of rickets or Fanconi syndrome (3). Several hormones influence serum phosphorus concentration, including parathyroid hormone (PTH); calcitriol, or 1,25-dihydroxyvitamin D_3_ (1,25(OH)_2_D_3_); and fibroblast growth factor 23 (FGF-23). PTH stimulates renal phosphate excretion, and calcitriol increases the absorption of phosphate by the intestine and bones. FGF-23 is a phosphaturic hormone that suppresses phosphate reabsorption in the kidneys, inhibits the synthesis of 1α-hydroxylase (CYP27B1), and increases the expression of 24-hydroxylase (CYP24A1), which controls calcitriol degradation and suppresses PTH production and secretion (2–4).

The three most common causes of hypophosphatemia are the intestinal malabsorption of phosphorus, the redistribution of tissue fluid phosphorus into cells, and an increased renal tubular loss of phosphorus. Table 1 shows the causes of renal phosphorus losses (3,5).

**Table 1:** Causes of renal phosphate wasting disorders

Disease	Etiology
Tumor-induced osteomalacia	Mesenchymal tumor producing FGF-23
Fibrous dysplasia	GNAS mutation
Cutaneous skeletal hypophosphatemic syndrome	Excess FGF23 production
Osteoglophonic dysplasia	FGFR1 mutation
NPHLOP1	SLC34A1 mutation
NPHLOP2	SLC9A3R1 mutation
Fanconi syndrome	SLC34A1 mutation
Raine syndrome	FAM20C mutation
Genetic hypophosphatemic rickets	
X‐linked hypophosphatemia (most common)	PHEX gene mutation
Autosomal dominant hypophosphatemic rickets	FGF23 gene mutation
Hereditary hypophosphatemic rickets with hypercalciuria	SLC34A3 mutation
Autosomal recessive hypophosphatemic rickets type 1	DMP1 mutation
Autosomal recessive hypophosphatemic rickets type 2	ENPP1 mutation
Hypophosphatemic rickets and hyperparathyroidism	α‐KLOTHO translocation
X‐linked recessive hypophosphatemic rickets	CLCN5 mutations
Dent disease 1	CLCN5 mutations
Dent disease 2	OCRL mutations

FGF-23: fibroblast growth factor 23; NPHLOP: hypophosphatemic nephrolithiasis/osteoporosis syndrome. Adapted from: Ruppe and Jan de Beur, 2018 (5).

This article will revise the novel treatment of two conditions that lead to hypophosphatemia with high FGF-23 levels: tumor-induced osteomalacia (TIO) and X-linked hypophosphatemic rickets (XLH).

## TUMOR-INDUCED OSTEOMALACIA AND X‐LINKED HYPOPHOSPHATEMIC RICKETS

TIO, also known as oncogenic osteomalacia, is a rare paraneoplastic syndrome characterized by excess production by tumors of FGF-23 and rarely other phosphatonins such as frizzled-related protein-4, fibroblast growth factor-7 (FGF-7), and matrix extracellular phosphoglycoprotein (MEPE), leading to hypophosphatemia, muscle weakness, and fractures (5–7). This condition is most common after the sixth decade of life. Most tumors are small, benign, and of mesenchymal origin (8). TIO is a rare condition that is difficult to diagnose, with a delay of approximately 2.5 years between the onset of symptoms and the diagnosis. Laboratory tests show reduced phosphorus, phosphaturia (secondary to reduced renal tubular phosphate proximal reabsorption), and low 1,25(OH)_2_D_3_, with normal calcium and PTH and markedly increased FGF-23 (5,9).

If hypophosphatemia is present, phosphaturia should be confirmed with a 24-hour urine sample. It can be evaluated by calculating the percentage of tubular phosphate reabsorption using the formula *TRP(%) = 1- [PO_4_ (Urine) × Creatinine (Plasm)]/ [PO_4_(Plasm) × Creatinine (Urine)] × 100*. Attention must be paid to the need for uniformity in all electrolyte units (6).

In patients who are unable to collect a 24-hour urine sample, a fractional excretion (FE) of filtered phosphate in a random urine sample calculated with the formula *FEPO_4_ = (Urine PO_4_ × Creatinine)/(Plasm PO_4_ × Urine Creatinine)* can be used. In patients with hypophosphatemia, an FE greater than 5% or a 24-hour urinary phosphorus greater than 100 mg is indicative of increased renal tubular phosphate loss (10).

With a major reduction in phosphorus and 1,25(OH)_2_D_3_, there is intense bone demineralization with severe osteomalacia at histomorphometry, increased mineralization lag time, and excessive osteoid (11).

The curative treatment for TIO is localization and resection of the tumor. However, the tumors are small and could take years to locate; in the meantime, the administration of phosphate and calcitriol is essential (11,12).

XLH is caused by inactivating mutations in the PHEX gene (phosphate regulator gene with homologies with endopeptidases on the X chromosome). The inactivation of PHEX mutations results in increased synthesis and secretion of FGF-23, causing phosphaturia, hypophosphatemia, and inappropriately low concentrations of 1,25(OH)_2_D3 (5,13). Children show progressive deformity of the lower limbs, decreased growth velocity, bone pain, and dental abnormalities. In adults, short stature, bone pain, deformities, and calcification of ligaments and tendons are present. Laboratory tests are sames of TIO (11).

Different molecular mechanisms – overproduction of FGF-23 by tumors responsible for oncogenic osteomalacia, generation of an FGF-23 mutant that is resistant to cleavage by enzymes, and impaired FGF-23 degradation due to the reduction or loss of PHEX – can lead to FGF-23-stimulating activity and the consequent waste of urinary phosphate (13).

## CONVENTIONAL TREATMENT FOR HYPOPHOSPHATEMIA DUE TO TIO OR XLH

The conventional treatment consists of multiple daily doses of oral phosphate salts and vitamin D metabolites or analogs as replacement therapy, with a phosphorus dosage of 20 to 40 mg/kg/day administered three to five times daily and a calcitriol dosage of 20 to 30 ng/kg/day. The treatment generally starts at a low dose to avoid gastrointestinal side effects and progressively increases until the improvement of symptoms and serum phosphorus (5). Conventional treatment leads to the resolution of radiographic rickets and improves but does not normalize growth, so some patients still need surgery to correct deformities. After the patient reaches adulthood, bone turnover decreases, and the closure of the epiphyseal plates makes the need for treatment variable. Some are not treated or need only a low dose of calcitriol alone, rarely are both calcitriol and phosphorus needed. Complications of conventional long-term treatment with calcitriol and phosphorus consist of hypercalcemia, hypercalciuria, nephrolithiasis, nephrocalcinosis, impaired renal function, and potentially chronic kidney disease (14).

Recently, the treatment of two conditions associated with an increase in FGF-23, XLH and TIO, has changed. Regulatory agencies have approved burosumab, an antibody against FGF-23, as a novel treatment for these conditions, presenting a new possibility beyond the conventional therapy (15–17).

## PHOSPHORUS AND FGF-23 PHYSIOLOGY

Phosphorus, an important mineral ion that is routinely consumed through food, is usually associated with oxygen in the form of phosphate. Phosphate is widely distributed in the body and is important in bone mineralization. The physiological phosphate balance is maintained by the coordinated interactions of the small intestine, bones, parathyroid glands, and kidneys, so functional deficiencies in any of these organs can lead to abnormal phosphate levels (2,3,18).

The serum phosphate concentration varies with age, with the highest concentration being in infants [normal range 4.5-8.3 mg/dL (1.50-2.65 mmol/L), conversion factor 0.322], who require more of the mineral for bone growth and soft tissue buildup. The concentration declines toward adulthood [normal range 2.5-4.5 mg/dL (0.8-1.5 mmol/L)] (2). Up to 70% of dietary phosphate can be absorbed by the upper half of the intestine, and the rest is excreted through urine. Intestinal cells may have a “phosphate sensor” because in studies with intestinal phosphate administration, phosphaturia occurs without the elevation of serum phosphorus. This intestinal regulation is unrelated to PTH, as studies in parathyroidectomized animals have shown (18,19). The transport of phosphate in the intestine (via enterocytes) and in the kidney (via proximal epithelial cells) is mainly mediated by proteins of the sodium-dependent phosphate transporter (NaPi) family (NaPi-2a, NaPi-2b, and NaPi-2c), which are expressed on the apical membrane of epithelial cells. In the kidneys, more than 80% of the filtered phosphate is reabsorbed in the proximal tubules via NaPi-2a and NaPi-2c (1,18,20).

Several endocrine factors, including parathyroid hormone, active metabolites of vitamin D, and FGF-23, can directly or indirectly control NaPi activities to influence the phosphate-balance system. PTH is one of the most potent regulators of phosphate metabolism. The parathyroid hormone can suppress phosphate reabsorption in the proximal tubules by reducing NaPi-2a and NaPi-2c, can mobilize phosphate from bones into the bloodstream, possibly increasing osteoclastic bone resorption, and can increase the production of 1,25(OH)_2_D_3_ by inducing the renal expression of 1-α hydroxylase, which affects intestinal phosphate absorption. In humans, dietary inorganic phosphate (Pi) supplementation increases FGF-23, and Pi restriction or the addition of Pi binders suppresses serum FGF-23 (21).

FGF-23 is considered a hormone because it is produced and secreted in bones by osteocytes, but its main target tissues are the distal nephron and the parathyroid gland, where both components of the FGF-23 receptor complex – FGF 1c receptor (FGFR1c) and membrane-bound αKlotho – are expressed. The secretion of FGF-23 by osteocytes is stimulated by a higher dietary phosphate intake or a lower excretion of phosphate in the glomerulus. Both parathyroid hormone and active vitamin D promote FGF-23 secretion and, by negative feedback, are suppressed by it (22). FGF-23 can suppress the expression of NaPi-2a and NaPi-2c cotransporters or the effect of parathyroid hormone activity, which induces urinary phosphate excretion. FGF-23 can also influence the vitamin D activity system by suppressing the renal expression of 1α-hydroxylase, which results in decreased calcitriol production. This induces parathyroid hormone secretion and concomitant phosphaturia (4,18).

Genetic and acquired abnormalities in the FGF23 structure and metabolism cause hyper-FGF-23 conditions – manifested by hypophosphatemia, low serum calcitriol, and rickets/osteomalacia.

## MECHANISM OF ACTION OF BUROSUMAB

Burosumab is a recombinant fully human immunoglobulin G monoclonal antibody developed for conditions with excess FGF-23. By binding to FGF-23, burosumab inhibits FGF-23 signaling, thereby increasing tubular phosphate reabsorption, decreasing renal phosphate excretion, increasing serum 1,25(OH)_2_D_3_ levels, and increasing the gastrointestinal absorption of phosphate. As a result, serum phosphate levels increase, ultimately bone mineralization is improved, and disease risk is reduced (16).

## BUROSUMAB STUDIES IN CHILDREN WITH XLH

In an open-label phase 2 study, 52 children between 5 and 12 years of age with XLH received subcutaneous burosumab every 2 or 4 weeks. They were assessed for symptom severity using the rickets severity score. There were decreases from 1.9 at the baseline to 0.8 at week 40 with dosing every 2 weeks and from 1.7 to 1.1 at week 40 with dosing every 4 weeks (p < 0.001 for both comparisons); these improvements persisted through week 64. The mean serum phosphorus level increased after the first dose in both groups, and more than half of the patients in both groups had levels within the normal range and remained within the reference until week 64 (with dosing every 2 weeks). Renal tubular phosphate reabsorption increased from the baseline in both groups. In both groups, the mean serum alkaline phosphatase level decreased. The patients’ physical abilities improved, and their pain decreased. The main adverse events were mild to moderate injection site reactions (15).

The extension of this study evaluated burosumab’s safety and efficacy for 160 weeks in children aged 5 to 12 years with XLH. Initially, burosumab was given every 2 weeks for one group and every 4 weeks for the other. After week 48, both groups received burosumab every 2 weeks. Of the 52 patients who completed the study, 41 children had open growth plates (79% from both treatment arms). The total rickets severity score decreased significantly from +1.57 ± 0.1 at week 64, +1.75 ± 0.1 at week 88, and +1.89 ± 0.1 at week 160 (p < 0.0001), and the increases in serum phosphorus were sustained over the 160 weeks. The safety profile was maintained with mild and moderate application-related side effects (23).

In a multicenter phase 3 study, children aged 1-12 years with XHL were included. Patients were randomly assigned (1:1) to receive either subcutaneous burosumab starting at 0.8 mg/kg every 2 weeks (burosumab group, 29: 16 girls, 13 boys) or a conventional therapy prescribed by the investigators (conventional therapy group, 32: 18 girls, 14 boys) for 64 weeks. Patients in the burosumab group had significantly greater improvement in the overall impression of their Global Radiographic Change scores than patients in the conventional therapy group. In these patients, the fasting serum phosphorus concentrations rose to within the normal range and remained normal throughout the trial. In contrast, the conventional therapy resulted in a slight increase in the fasting serum phosphorus level and a slight decrease in the tubular maximum phosphate/glomerular filtration rate (TmP/GFR). The burosumab treatment also resulted in greater significant increases in the serum 1,25(OH)_2_D_3_ level compared to the conventional therapy. One serious adverse event occurred (tooth abscess) but was not considered treatment-related. In the burosumab group, 45% of patients had injection site reactions, none of which were severe (24).

In another phase 2 study at three hospitals in the US, 13 children (aged 1-4 years) with XHL received burosumab (0-8 mg/kg) via subcutaneous injections every 2 weeks for 64 weeks. The Thacher rickets total severity score decreased, and the Radiographic Global Impression of Change score (RGI-C score) also indicated significant improvement after the burosumab was administered. Treatment with burosumab for 40 weeks resulted in a significant improvement of rickets. All treated patients had overall RGI-C scores ≥ +2 (7-item scale), ranging from -3 (severe worsening) to +3 (substantial improvement/cure). The mean RGI-C scores at week 40 were +2.3 ± 0.08 for the global assessment, +2.3 ± 0.11 for the wrist assessment, and +2.2 ± 0.15 for the knee assessment (p < 0.0001). The maximum values for the RGI-C score for global, wrist, and knee were +2.7, but no patient had a score of +3, which would indicate a complete or near complete reversal of rickets. All patients had at least one adverse event. Fourteen treatment-related adverse events, mostly injection site reactions, occurred in five children. One serious adverse event considered unrelated to treatment (dental abscess) occurred in one child with a history of dental abscesses (25).

More recently, a real-life retrospective study evaluated 12 patients aged 1-18 years with XLH who were previously treated with conventional therapy and transferred to burosumab. After 1 month of the burosumab treatment, their phosphorus increased; their alkaline phosphatase, PTH, and urinary phosphorus excretion decreased; and these improvements were maintained for 2 years. The rickets severity score and height Z scores also improved. There were no serious adverse events with the burosumab treatment (26). Table 2 summarizes the main studies on burosumab treatment in children.

**Table 2:** Main studies on burosumab in children

Studies in children	Trial design	Primary outcome	Comparator and/or intervention	Results
Carpenter and cols., 2018 (15)	Phase 2, open-label, 5- to 12-year-old XLH patients exposed to 2 treatment regimens with burosumab: 0.2 to 0.3 mg/kg every 2 weeks, or 0.4 to 0.6 mg/kg every 4 weeks for 64 weeks.	Thacher rickets severity score (0 to 10: 0 less severity and 10 high severity) at weeks 40 and 64.	52 patients: 26 patients received 0.2 to 0.3 mg/kg burosumab every 2 weeks; 26 patients received 0.4 to 0.6 mg/kg burosumab every 4 weeks.	Thacher rickets severity total score decreased from 1.9 at baseline to 0.8 at week 40 with every 2-week dosing and from 1.7 at baseline to 1.1 at week 40 with every 4-week dosing (p < 0.001 for both); these improvements persisted at week 64.
Imel and cols., 2019 (24)	Phase 3, randomized, active-controlled, open-label, multicenter with 1- to 12-year-old XLH patients for 40 weeks.	Change in rickets severity at week 40, assessed by the Radiographic Global Impression of Change global score.	61 patients: 32 (18 girls, 14 boys) received conventional therapy; 29 (16 girls, 13 boys) received burosumab starting at 0.8 mg/kg every 2 weeks until 64 weeks.	At week 40, patients in the burosumab group had significantly greater improvements in GRCS than did patients in the conventional therapy group (least squares mean +1.9 [SE 0.1] with burosumab vs +0·8 [0.1] with conventional therapy; difference, 1.1, 95% CI 0.8-1.5; p < 0.0001).
Whyte and cols., 2019 (25)	Phase 2, open-label, multicenter, children (aged 1-4 years) with XLH received burosumab (0.8 mg/kg) every 2 weeks for 64 weeks.	Safety and change from baseline to week 40 in fasting serum phosphorus concentrations.	13 children completed 64 weeks of treatment.	Serum phosphorus least squares mean increase from baseline to week 40 of treatment was 0.31 mmol/L (SE 0.04; 95% CI 0.24-0.39; 0.96 mg/dL [SE 0.12]; p < 0.0001). All patients had at least one adverse event, mostly injection site reactions.

XLH: X-linked Hypophosphatemia; SE: standard error; GRC: Global Radiographic Change Score; CI: confidence interval.

## BUROSUMAB STUDIES IN ADULTS WITH XLH

The largest study evaluating burosumab treatment in adults with XLH was published in two phases (24-week and the extension at 48-week follow-up). CL-303 is a phase 3, randomized, double-blind, placebo-controlled, multicenter clinical trial that evaluated the efficacy and safety of burosumab in adult patients (18 to 65 years old) with XLH. The study included 134 patients (burosumab group = 68; placebo group = 66). About 94% of patients in the burosumab group achieved serum phosphorus levels above the lower limit of normal at the midpoints of the dose intervals by week 24, compared to only 7.6% of patients in the placebo group (p < 0.0001). In this group, the increase in serum phosphorus concentrations normalized at week 1 and was maintained throughout the treatment. At week 24, 43.1% (burosumab group) and 7.7% (placebo group) of the patients’ basal active fractures were fully healed; the chance to have a consolidated fracture was 16.8 times higher in the burosumab group than in the placebo group (p < 0.001) (27).

The trial was extended to week 48, with patients on the placebo being treated with burosumab and those already on burosumab continuing their treatment. This extension demonstrated that the continued treatment with burosumab was well tolerated, leading to sustained correction of serum phosphorus levels, continued healing of fractures and pseudofractures, and sustained improvement of major musculoskeletal impairments (28).

Adverse effects were reported in almost all adults receiving burosumab or the placebo in the first 24 weeks of the trial (94.1% and 92.4%, respectively). The most common were back pain (15% and 9%), dental abscess/infection (13% and 8%), headache/head discomfort (13% and 8%), restless leg syndrome, and dizziness (10% and 6%), with all percentages representing the burosumab and placebo recipients, respectively. Although serious effects were reported, none of them were considered related to the studied drug (27,28).

An extension of the studies cited above has recently been published. In this 96-week extension, patients achieved significant improvements in their pain inventory (BPI-SF), brief fatigue inventory (BFI), and stiffness (WOMAC) scores. The improvements in the 6-minute walking distance test and the predicted percentage were significant from week 24 to 96 (29).

The bone histomorphometry of 11 patients was evaluated before the burosumab treatment and after 48 weeks of treatment at a dose of 1 mg/kg every 4 weeks. All osteomalacia-related histomorphometric measures improved significantly at week 48 (mean percentage change: osteoid volume/bone volume, -54%; osteoid thickness, -32%; osteoid surface/bone surface, -26%; mineralization delay time [median], -83%). Bone formation and resorption markers increased at week 48 (least significant [LS] mean increase): type 1 procollagen amino-terminal propeptide (P1NP), +77% (52.5 ± 11.6 ng/mL) and carboxy-terminal collagen crosslinks (CTX), +36% (175.1 ± 44.0 pg/mL) (both p < 0.0001) (30). Table 3 summarizes the main studies on burosumab treatment in adults.

**Table 3:** Main studies of burosumab in adults

Study in Adults	Trial design	Primary outcome	Comparator and/or intervention	Results
Insogna and cols., 2018 (27)	Phase 3, randomized, double-blind, placebo-controlled, multicenter in adult patients (18 to 65 years old) with XLH for 24 weeks.	Proportion of subjects achieving a mean serum phosphate concentration above the LLN of 2.5 mg/dL (0.81 mmol/L).	134 patients (burosumab group = 68; placebo group = 66).	94.1% of participants in the burosumab group versus 7.6% in the placebo group achieved a mean serum phosphate concentration above the LLN (p < 0.001).
Portale and cols., 2019 (28) (Extension of Isogna and cols., 2018)	Phase 3, randomized, double-blind, placebo-controlled, multicenter in adult patients (18 to 65 years old) with XLH for 48 weeks.	Compared the efficacy of burosumab and placebo through week 24.	134 patients, by week 24, 68 patients were in the burosumab group and 66 in the placebo group. After week 24 until week 48, all subjects received burosumab.	In weeks 24 to 48, phosphorus concentrations remained normal in 83.8% of participants who received burosumab and were normalized in 89.4% of participants who received burosumab after the placebo.
Briot and cols., 2021 (29) (Extension of Isogna and cols., 2018)	Phase 3, randomized, double-blind, placebo-controlled, multicenter in adult patients (18 to 65 years old) with XLH for 96 weeks.	Effect of burosumab on physical function, stiffness, pain, and fatigue (at weeks 24, 48, and 96) and ambulatory function (at weeks 24, 48, and 72) compared with baseline.	134 patients: by week 24, 68 patients were in the burosumab group and 66 were in the placebo group. After week 24 until week 96, all subjects received burosumab.	At week 96, all patients achieved a statistically significant improvement in pain inventory (BPI-SF), BFI, and stiffness (WOMAC) scores. Improvements in the 6-minute walking distance test and the predicted percentage were statistically significant from weeks 24 to 96.
Insogna and cols., 2019 (30) (Extension of Isogna and cols., 2018)	Phase 3, randomized, double-blind, placebo-controlled, multicenter in adult patients (18 to 65 years old) with XLH for 48 weeks.	Improvement in osteoid volume/bone volume assessed by trans iliac bone biopsies obtained at baseline and week 48.	13 completed 48 weeks, and 11 completed paired biopsies.	All osteomalacia-related histomorphometric measures improved significantly at week 48 (mean percent change: osteoid volume/bone volume, –54%; osteoid thickness, –32%; osteoid surface/bone surface, 26%; [median] mineralization lag time, 83%).

XLH: X-linked Hypophosphatemia; LLN: lower limit of normal; BFI: brief fatigue inventory; WOMAC: Western Ontario and the McMaster Universities Osteoarthritis Index stiffness subscale.

## BUROSUMAB STUDIES IN ADULTS WITH TIO

Studies on the treatment of tumor-induced osteomalacia are scarce due to the rarity of the cases. A phase 2, open-label, single-arm study evaluated the efficacy and safety of burosumab in adults (aged ≥18 years) with TIO not curable by surgical excision or with cutaneous hypophosphatemia syndrome (CSHS), a rare condition defined by the association of epidermal and/or melanocytic nevi, a mosaic skeletal dysplasia, and FGF23-mediated hypophosphatemia. (31). The co-primary endpoints were the proportion of patients with mean serum phosphorus levels above the lower limit of normal (>0.81 mmol/L) at week 24 and changes in osteomalacia parameters at week 48, as assessed by osteoid thickness, osteoid surface/bone surface area, osteoid volume/bone volume, and delayed mineralization time. Seven (50%) patients achieved mean serum phosphorus levels above the lower limit of normal (0.81 mmol/L). At week 48, most osteomalacia-related histomorphometric measures improved with the burosumab treatment (32).

In conclusion, phosphorus is a mineral required to maintain bone integrity and mineralization. Increased renal tubular loss of phosphorus is a cause of hypophosphatemia; it could be due to a tumor producing FGF-23 or a genetic disorder (XLH) caused by an inactivating mutation in the PHEX gene. The conventional treatment with oral phosphate salts and vitamin D metabolites or analogs partially improves rickets or osteomalacia caused by a lack of mineralization, and complications such as growth deficits, nephrocalcinosis, and decreases in kidney function can occur. Burosumab, a recombinant fully human monoclonal antibody that binds to FGF-23, inhibits FGF-23 signaling and could reverse the effects of excess FGF-23, promoting increased phosphate levels, improved bone mineralization, and reduced morbidity in both conditions, XLH and TIO.
